# Case report: Clinical and molecular features of renal gastrointestinal tumor

**DOI:** 10.3389/fonc.2025.1508600

**Published:** 2025-02-13

**Authors:** Simran Makker, Rayan Rammal, Ping Gu, Guido Dalbagni, Hikmat Al-Ahmadie, Narasimhan P. Agaram, Gopa Iyer, Ritesh R. Kotecha

**Affiliations:** ^1^ Laurel Springs School, Ojai, CA, United States; ^2^ Department of Pathology, Memorial Sloan Kettering Cancer Center, New York, NY, United States; ^3^ Department of Medicine, Memorial Sloan Kettering Cancer Center, New York, NY, United States; ^4^ Department of Surgery, Sloan Kettering Cancer Center, New York, NY, United States

**Keywords:** gastrointestinal stromal tumor, GIST, renal mass, imatinib, KIT

## Abstract

While gastrointestinal stromal tumors (GISTs) often arise within the GI tract, it is well known that GISTs may also rarely emanate outside of the digestive system. Prior case reports have documented various primary sites in non-GI organs [extra-intestinal GIST (EGIST)], yet only one report has described a localized GIST of renal origin. Here, we describe a patient who presented with bilateral renal masses who was found to have a large unresectable renal GIST tumor treated with imatinib. We discuss treatment experience and response with systemic therapy and describe molecular data to contextualize this ultra-rare presentation within the landscape of EGIST tumors.

## Introduction

Gastrointestinal stromal tumors (GISTs) represent the most common soft-tissue mesenchymal tumor within the GI tract. While these tumors usually arise within the stomach and small intestine, a subset of GISTs (<5%) originate in other organ sites (1), as detailed in several case reports (2–4). As most GIST tumors have identifiable driver alterations that characterize the natural history of these molecular subtypes and overall guide treatment strategy, studying EGISTs sheds light on common biology associated with tumorigenesis. To our knowledge, only one prior case report describes a patient diagnosed with a localized renal GIST who subsequently underwent nephrectomy and adjuvant imatinib therapy (5). Here, we present a patient diagnosed with bilateral renal masses found to have a large, unresectable, *KIT* exon 11-mutant, renal GIST tumor treated with imatinib. We discuss the clinical presentation, with emphasis on bilateral renal masses, treatment experience, and molecular findings to contextualize this rare tumor presentation in the landscape of EGIST tumors.

## Case presentation

**Figure 1:**
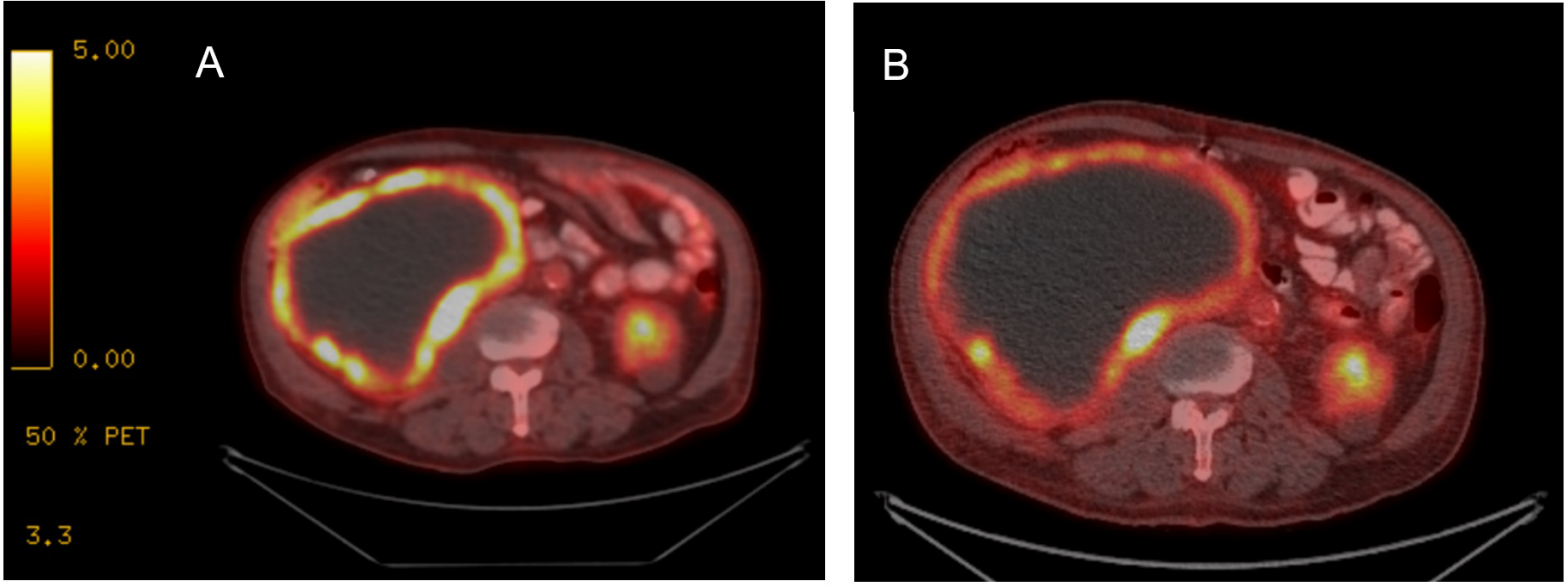
FDG PET/CT with treatment response to imatinib. **(A)** At diagnosis, FDG PET/CT imaging demonstrated a large right renal tumor with peripheral enhancement and necrosis. **(B)** After treatment initiation, while tumor size is comparably similar, peripheral avidity has decreased, consistent with overall treatment response.

A 78-year-old previously healthy man presented for medical care due to worsening abdominal pain and distension. He previously had been undergoing routine health examinations and prostate serum antigen monitoring for benign prostate hypertrophy and had no previously documented family history of cancer. At initial evaluation, he underwent an abdominal ultrasound that showed a large abdominal mass. A subsequent contrast-enhanced computed tomography (CT) abdomen/pelvis demonstrated a large right partially necrotic mass (16.8 × 12.7 cm) with associated compression of the proximal ureter and a largely necrotic 4.5 × 4.1 cm mid-left renal mass. He therefore sought care at our institution, where an F-fluorodeoxyglucose (FDG) positron emission tomography (PET)/CT showed a peripherally hypermetabolic right renal mass measuring 17.6 × 12.9 cm with a maximum standardized uptake value (SUV) of 11.5 and a 5.2 × 4.8 cm mildly peripherally avid left renal mass (SUV 2.9) (**Figure 1A**). Laboratory data at presentation indicated normal renal function with a serum creatinine of 1.1 mg/dL and mild anemia (hemoglobin 11.0 g/dL) and a normal lactate dehydrogenase (145 U/L).

**Figure 2:**
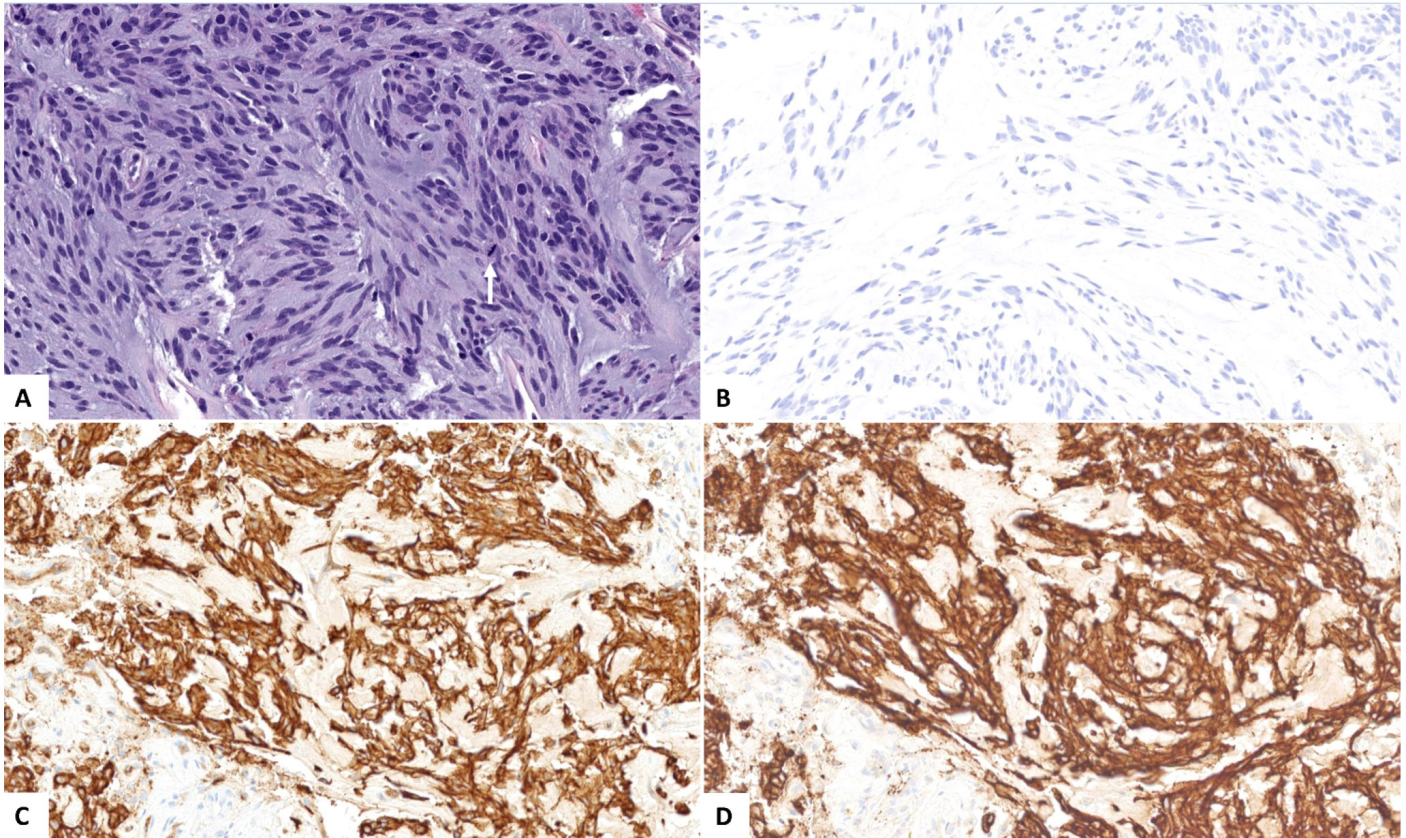
**(A)** Microscopic examination reveals a neoplastic proliferation of spindle cells arranged in fascicles and in a myxoid background, with mitotic figures (arrow, hematoxylin and eosin stain, 200× magnification). The tumor cells are negative for **(B)** PAX8 while diffusely and strongly positive for **(C)** CD117 and **(D)** DOG1 (200×).

**Figure 3:**
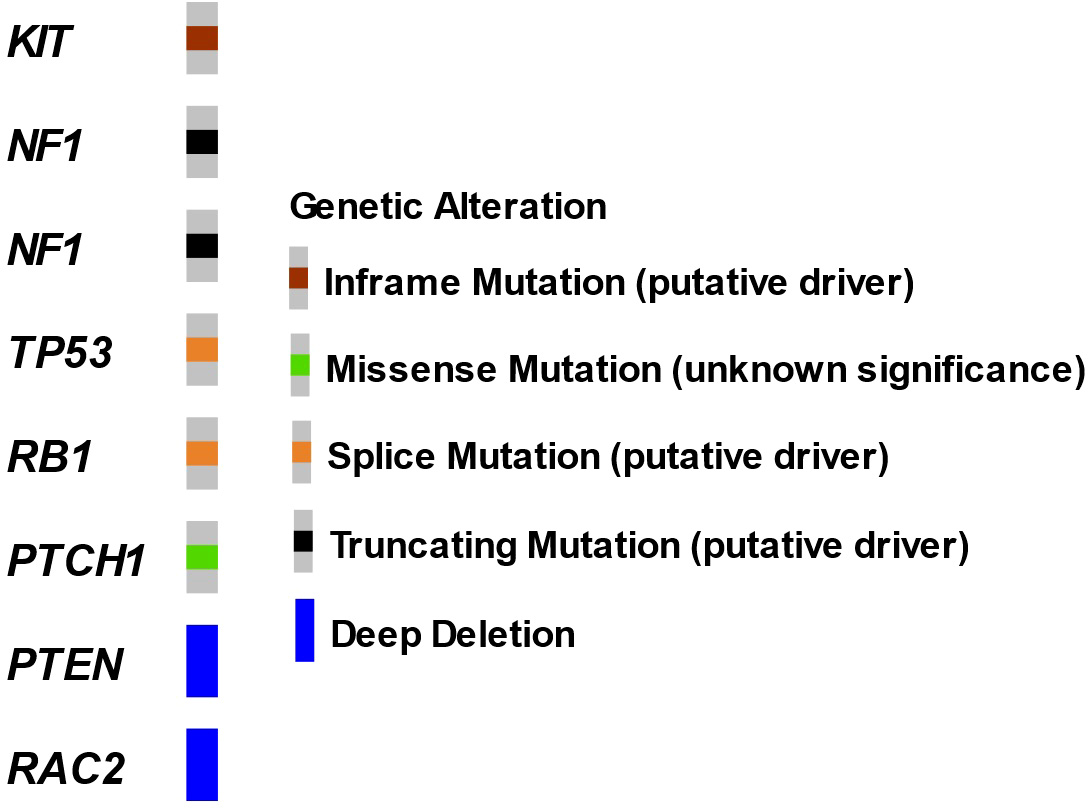
Oncoprint of renal GIST tumor by MSK-IMPACT. Next-generation sequencing performed on a core renal tumor biopsy specimen.

The patient underwent a CT-guided right renal core needle biopsy, and pathology demonstrated a GIST, spindle cell type, with spindle cells arranged in fascicles in a myxoid background. Tumor necrosis was present. By immunohistochemistry (IHC), the tumor showed strong, diffuse positivity for DOG1 (ANO1) and CD117, and negativity for SMA, desmin, HMB45, Mel-A, CDK4, MDM2, S100, CKAE1/AE3, CK7, racemase, and PAX8 (**Figure 2**). Expression of H3K27me by IHC was retained. The mitotic activity was 12 per 10 high-power fields, and the Ki-67 index was 15%. Next-generation targeted panel sequencing by MSK-IMPACT of the tumor specimen detected a *KIT* exon 11 mutation (D579del) and alterations in *NF1, TP53, RB1*, and *PTCH1*, with loss of *PTEN* and *RAC2* by copy number (**Figure 3**). Copy number analysis by FACETs suggested widespread loss of heterozygosity.

**Figure 4:**
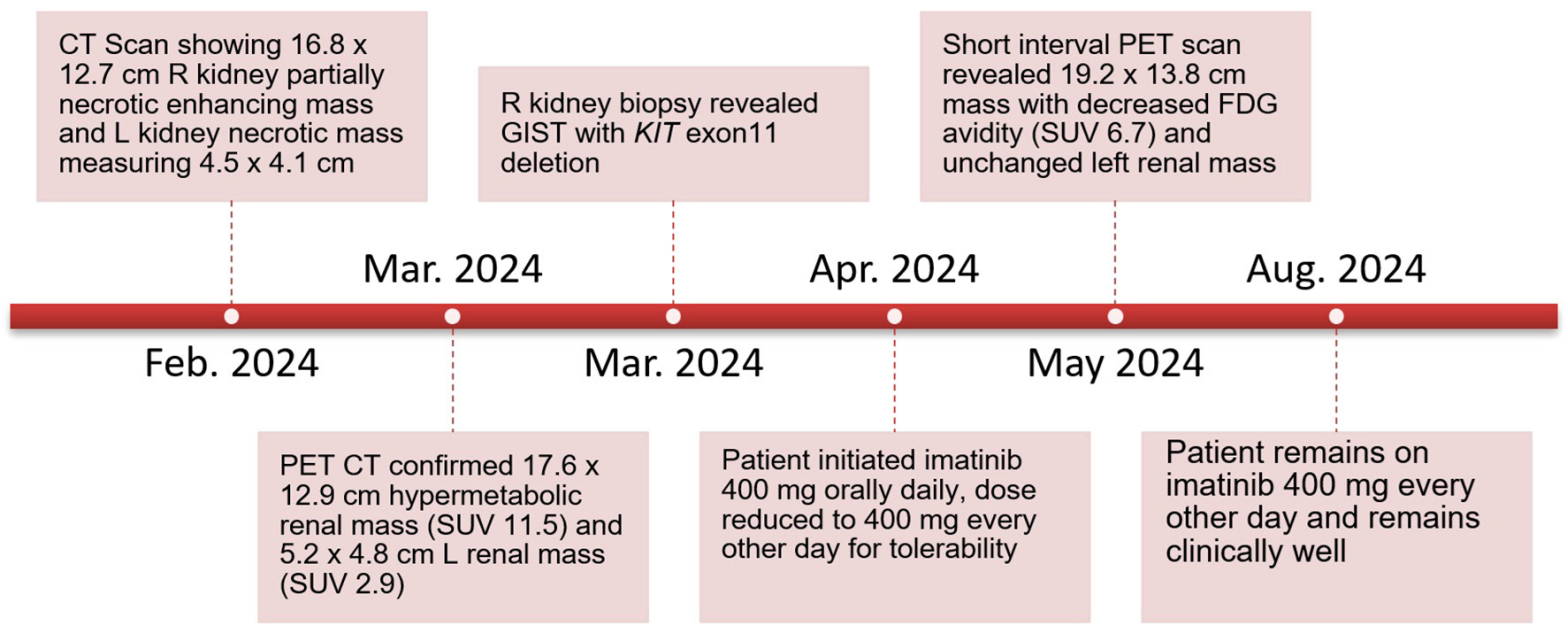
Patient disease course and follow-up.

The patient underwent evaluation by urology and medical oncology, and based on the presence of bilateral renal masses and the large unresectable nature of the proven GIST tumor, the left renal tumor was not biopsied as the results would not impact initial disease management. He was initiated on systemic therapy with imatinib 400 mg orally daily with subsequent dose reduction to 400 mg every other day for tolerability. A short-interval FDG PET/CT obtained 4 weeks after treatment initiation revealed that the peripherally hypermetabolic right GIST tumor was slightly larger (measured 19.2 × 13.8 cm) but had decreased hypermetabolic activity within the posterior medial wall of the mass (SUV 6.7), consistent with treatment effect (**Figure 1B**). The other peripherally avid left renal mass was similar in tumor measurement and avidity. He has continued imatinib with 6 months of follow-up given ongoing clinical benefit and follows up in the outpatient oncology clinic (**Figure 4**).

## Discussion

We report a case of bilateral renal masses initially suspicious for renal cell carcinoma (RCC) and ultimately found to be an unresectable renal GIST tumor. Although the presence of bilateral renal masses poses both diagnostic and therapeutic challenges, we present this case to highlight a rare presentation of an EGIST tumor. While the retroperitoneum remains a common site for EGIST tumors, which can lead to indirect invasion of the kidney, radiographic and pathologic findings in this case pointed towards a renal origin. In a previously reported renal GIST case (5), the patient had presented with a large solitary renal tumor accompanied by hydronephrosis. He underwent nephrectomy and was treated with adjuvant imatinib. In contrast, in our case, the patient was started on upfront systemic therapy with imatinib, and short-interval post-treatment PET/CT showed clinical response. Molecular analysis identified an oncogenic *KIT* alteration, which may portend sensitivity to tyrosine kinase inhibitor (TKI) therapy. To our knowledge, this is the first case report highlighting imatinib therapy with data showing tumor response for renal EGIST, and the first report showing molecular features associated with a renal EGIST tumor site.

GISTs are mesenchymal tumors that arise from interstitial cells of Cajal (ICC) that exhibit functional heterogeneity across different organ sites (6). ICC-like cells have been observed in other tissues including the cardiovascular system (7), lungs (8), skin (9), mammary glands (10), liver and biliary system (11), male and female reproductive systems (12), placenta (7), prostate (13), urinary bladder, renal pelvis, and uretero-pelvic junction (7, 13, 14). ICC-like cells can modulate immune response, regulate blood flow, and play a role in angiogenesis through the production of vascular endothelial growth factor (VEGF), extracellular matrix organization, and cell migration (10, 15), and harbor electrical capacity that can influence contractile activity (16). In the urinary system, these cells may conduct and amplify the pacemaker signals generated by atypical smooth muscle cells and modulate signal transduction (17) and may vary in density and distribution by location in the urinary system and in the setting of pathology (18).

Patients with bilateral renal masses pose both diagnostic and therapeutic challenges. In the diagnostic differential, these tumors may represent either a second primary or a metastasis from the contralateral kidney. In this case, biopsy of the small left renal tumor was deferred as immediate clinical management would not be impacted and followed clinically to ascertain etiology based upon concordant or discordant response to systemic therapy. Bilateral renal tumors are seen in up to 5% of patients with RCC, and in these situations, tumor resection is often limited by the need to preserve renal function postoperatively. In the context of bilateral tumors, hereditary syndromes, including von Hippel Lindau, are usually invoked, prompting germline testing (19). In this individual, germline testing did not detect a known pathogenic alteration with increased hereditary risk. Interestingly, Carney-Stratakis syndrome, characterized by germline mutations in *SDHB*, *SDHC*, or *SDHD*, is a hereditary syndrome associated with GIST and paraganglioma, and may also be associated with SDH-mutant RCC. Therefore, when evaluating patients with the potential for both entities, it is important to consider if there remains a disease overlap with rare presentations.

The presence of *KIT* or *PDGFRA* gain-of-function alterations are highly specific for GIST tumors, found in up to 85% of these tumors, and these alterations also guide treatment decision-making (20–22), with *KIT* mutations comprising 75%–80% of cases (23–25). In the remaining 15% of GISTs, alterations in a variety of genes have been reported, including *NF1, SDH, BRAF, KRAS, PIK3CA*, and *ETV6*::*NTRK3* gene fusion (26–31). Notably, this patient was found to have a co-mutation in *NF1* and deletion of *PTEN*. GISTs harboring *KIT* mutations, particularly those with exon 11 mutations, are associated with improved response to imatinib therapy. While prior reports have detailed that E-GIST tumors harbor similar molecular profiles to GIST tumors, *TP53* alterations, as seen in our patient, appear less commonly in conventional GIST and are usually found in more aggressive, advanced stage tumors. Outside of genomic features, elevated Ki67 (as seen in our case) also suggests higher risk disease and appears to be more typical in EGIST tumor sites.

FDG PET/CT is an essential tool for staging and early treatment response evaluation in GIST tumors. GISTs typically exhibit strong FDG uptake; moreover, the sensitivity and positive predictive value of PET/CT are 86% and 98%, respectively, with false-negative results mainly attributed to small lesions that are below PET scan resolution (32). PET/CT is also a useful imaging modality to assess early response to imatinib treatment as tumor size alone is unreliable for assessing response to TKI therapy treatment. GIST tumors may not change significantly in size and may even grow while responding to imatinib administration (33), and cystic and density changes can precede tumor shrinkage. One study demonstrated that the overall GIST disease burden evaluated according to changes in size, density, and number of tumor nodules and intralesional vasculature correlated best with the reduction of maximum SUV on FDG PET/CT scans (34). This was apparent initially during this case with reduction in avidity occurring soon after treatment initiation.

In sum, we present a case of bilateral FDG avid renal masses with subsequent identification of a renal GIST tumor. Treatment with imatinib yielded early and durable systemic therapeutic benefit, and molecular analysis revealed a *KIT* exon 11 mutation that portends favorable sensitivity to imatinib systemic therapy. This case highlights that renal GIST tumors have similar molecular profiles to conventional GIST tumors and systemic therapy approaches including precision medical therapies can similarly impact treatment response in this rare subtype.

## Data Availability

The original contributions presented in the study are included in the article/supplementary material. Further inquiries can be directed to the corresponding author.
